# Genetic and Molecular Regulation of Neuronal Excitability in Anesthesia and Pain

**DOI:** 10.3390/genes17101283

**Published:** 2026-10-15

**Authors:** Zheng Liu, Tong Wang, Xinran He, Siyuan Song

**Affiliations:** 1 Department of Pathology, The University of Texas MD Anderson Cancer Center, Houston, TX 77030, USA; zhengliu.jan@gmail.com; 2 Department of Biology, Duke University, Durham, NC 27708, USA; wangtongnelly@gmail.com; 3 Department of Anesthesiology, Boston University Chobanian & Avedisian School of Medicine, Boston, MA 02118, USA; xinran.he@bmc.org; 4 Department of Anesthesiology, Montefiore Medical Center, Albert Einstein College of Medicine, Bronx, NY 10467, USA

**Keywords:** neuronal excitability, anesthesia, nociceptors, ion channels, neuroimmune signaling, human genetics

## Abstract

Genetic and molecular studies increasingly explain anesthetic and analgesic responses at the level of defined conductances, neuronal populations, and injury states. Their clinical implications depend on distinguishing target engagement from changes in firing and, ultimately, from a measurable therapeutic effect. This review examines recent evidence linking sodium, potassium, calcium, and sensory transduction channels to pain and inhibitory receptor composition, chloride regulation, and arousal circuits to anesthesia. Selective NaV1.8 inhibition provides an unusually complete translational example, spanning channel pharmacology, recordings from human sensory neurons, and randomized postoperative trials. Nevertheless, residual firing during substantial NaV1.8 inhibition and divergent explanations of NaV1.7-null analgesia show why the effects of a drug cannot be inferred directly from a genetic phenotype. Structural studies now resolve anesthetic binding and gating mechanisms in GABAA, NMDA, and HCN1 channels, while circuit experiments distinguish emergence, immobility, and antinociception. Neuroimmune signaling extends beyond cytokine-driven excitation to include inhibitory synapse removal, sex-dependent cellular pathways, and active pain resolution. Chromatin regulation, RNA modification, and neuronal senescence can stabilize altered excitability after injury. Human ganglion and spinal cord atlases, Patch-seq, and sensory assembloids provide more relevant models for connecting these mechanisms across scales, although none substitute for clinical validation. We emphasize research published from 2021 onward, retaining foundational studies where they establish a causal mechanism. The resulting evidence supports cell- and state-specific intervention rather than a single molecular explanation of anesthesia or chronic pain and identifies practical requirements for translating genetic discovery into perioperative treatment.

## 1. Introduction

Anesthesia and pain involve overlapping electrical machinery, but they are not opposite positions on one scale of neuronal activity. An anesthetic can suppress coordinated cortical responses while leaving substantial sensory transmission intact. Conversely, an analgesic can reduce nociceptive output without impairing consciousness. Recent experiments make this separation concrete: selective NaV1.8 inhibition reduces postoperative pain, the stimulation of defined thalamic neurons restores arousal during propofol administration, and the activation of an anesthetic-recruited amygdala ensemble suppresses nocifensive behavior. Each intervention acts at a different biological level and requires a different outcome measure [1,2,3].

Excitability is correspondingly multidimensional. Resting membrane conductance, the action potential threshold, repetitive firing, axonal propagation, and transmitter release can change independently. A channel inhibitor may narrow the action potential without abolishing its initiation; reduced potassium recruitment can even shorten the refractory period. Human dorsal root ganglion (DRG) recordings during suzetrigine exposure demonstrate precisely this combination, challenging the assumption that stronger channel inhibition always produces a proportionate reduction in firing [4]. At the circuit level, suppressing an inhibitory neuron can increase downstream activity, whereas activating an inhibitory projection can produce analgesia. The molecular mechanism therefore requires a specified cell type, anatomical compartment, and physiological measurement.

Human genetics established the importance of this approach before the current wave of molecular profiling. The biallelic *SCN9A* loss of function causes profound pain insensitivity, while selected gain-of-function variants produce painful syndromes through altered NaV1.7 gating [5,6]. More recently, a chronic pain intensity association at *SLC45A4* was connected to polyamine transport, protein structure, and reduced nociceptor excitability in knockout mice [7]. These examples differ from a gene expression association because they connect molecular variation to an experimentally testable function. Neither, however, guarantees that an adult pharmacological intervention will reproduce a lifelong genotype.

This narrative review emphasizes original studies published from 2021 to September 2026, with selected earlier experiments retained where they establish causal principles. We first examine membrane and synaptic mechanisms, then consider anesthetic circuits, neuroimmune regulation, and persistent changes in gene expression. Human cellular models and clinical trials are used to assess which mechanisms have progressed beyond target nomination. The aim is to explain how recent findings revise earlier models, including where results disagree, rather than compile an undifferentiated list of pain- or anesthesia-associated genes.

## 2. Membrane Conductances and the Molecular Control of Sensory Output

### 2.1. Sodium Channel Genetics Now Meet Human Pharmacology

NaV1.7 and NaV1.8 contribute different but interacting components of sensory electrogenesis. NaV1.7 amplifies depolarization near rest, whereas NaV1.8 can support the action potential and repetitive firing at more depolarized voltages. In dynamic clamp experiments modeling the erythromelalgia-associated NaV1.7 L848H variant, subtracting 25–50% of NaV1.8 conductance increased rheobase and reduced firing probability. The important finding is the interaction between conductances, not a universal inhibition threshold applicable to every nociceptor [8]. The earlier identification of gain-of-function *SCN10A* variants in painful neuropathy provided human genetic support for NaV1.8, but pathogenicity still requires variant-specific functional and clinical evidence [9].

Suzetrigine has made the distinction between molecular selectivity and neuronal efficacy experimentally accessible. Recombinant channel studies reported at least 31,000-fold selectivity over other NaV subtypes and binding to the second voltage-sensing domain, stabilizing a closed channel conformation [10]. LTGO-33 acts at a related extracellular voltage-sensor region and inhibits human DRG firing but exhibits marked primate-versus-rodent pharmacological differences [11]. Thus, selecting a nominally conserved channel does not remove the need to establish species-specific compound potency. The voltage-sensor mechanism also differs from the pore-associated binding underlying many traditional sodium channel blockers.

State dependence illustrates why assay design can change the apparent mechanism. Initial experiments showed the relief of VX-548 inhibition during strong repetitive depolarizations, described as reverse use dependence [12]. Subsequent comparisons using action potential waveforms at 37 °C found essentially no accumulated relief for suzetrigine or LTGO-33 during physiological stimulus trains. Suzetrigine dissociated too slowly during brief spikes; LTGO-33 rapidly re-bound to the channels between them [13]. These results are compatible once voltage, duration, and temperature are considered. A depolarizing square pulse that reveals a binding transition does not necessarily predict drug behavior during a nociceptor action potential.

Human neuronal recordings add another constraint. At concentrations substantially above the recombinant channel IC50, suzetrigine reduced action potential peaks and shoulders more than the initial threshold or upstroke velocity. Repetitive firing was usually reduced but not eliminated, partly because a small remaining fraction of a large NaV1.8 current can remain physiologically substantial and because NaV1.7 contributes to spike generation. Some cells fired faster as their refractory period shortened [4]. Dynamic clamp and human pharmacology studies therefore answer different questions: reducing one conductance can correct a particular hyperexcitable state without guaranteeing electrical silence across heterogeneous adult human neurons.

NaV1.7 remains a strong example of the difficulty of reproducing genetic analgesia pharmacologically. Earlier work implicated increased endogenous opioid signaling in NaV1.7-null analgesia [14]. A 2023 study instead found enkephalin upregulation restricted to C low-threshold mechanoreceptors and demonstrated the opioid-independent failure of nociceptor action potential initiation [15]. A 2024 comparison distinguished embryonic from adult deletion and reported compensatory increases in other sodium channel proteins after embryonic loss [16]. These findings should be presented as an unresolved, model-dependent mechanistic debate. Developmental timing, the cells in which deletion occurs, recording location, and autonomic effects all influence the interpretation; one knockout phenotype cannot serve as a universal prediction of acute channel blockade.

Several recent approaches target channel regulation rather than the pore. Disrupting a NaV1.7-specific CRMP2 interaction sequence reduced trafficking, spinal CGRP release, and neuropathic hypersensitivity in rodents; an AAV-encoded construct also reduced channel function in rhesus sensory neurons [17]. TMEM233 was identified as a sensory-neuron accessory protein required for the action of a stinging-tree toxin and capable of modifying NaV1.7 gating [18]. In patient-derived sensory neurons, the correction of the *SCN9A* A1632G variant reduced hyperexcitability, while introduction into a control line reproduced it, providing a particularly informative isogenic causal test [19].

Target localization can also change the biological interpretation. NaV1.7 deletion in sensory neurons reduced osteoarthritis pain, whereas its deletion in chondrocytes affected structural disease progression, demonstrating distinct functions of the same channel in different cells [20]. Follistatin released after nerve injury increased nociceptor excitability through IGF1R signaling, with effects demonstrated in human DRG neurons as well as mice [21]. Separately, the paracetamol metabolite AM404 was reported to inhibit NaV1.7 and NaV1.8 through the local anesthetic binding region in peripheral neurons [22]. This is mechanistic evidence for an additional peripheral action, not proof that it accounts for all clinical acetaminophen analgesia.

Follistatin experiments are particularly useful for distinguishing expression from intercellular signaling. Injury increased follistatin in A-fiber neurons, whereas its excitability-promoting effect involved nociceptive neurons. Binding assays and coimmunoprecipitation supported interaction with IGF1R, and receptor inhibition reduced ERK/AKT activation and hyperexcitability. A peptide interfering with the interaction attenuated NaV1.7-associated responses [21]. The proposed mechanism therefore includes a source population, extracellular mediator, recipient receptor, and electrical output. This is more specific than attributing pain to increased growth-factor signaling, and it suggests experiments that distinguish altered mediator release from changed receptor sensitivity in the recipient neuron.

### 2.2. Potassium and Calcium Channels Regulate Different Electrical Operations

Potassium channels determine the membrane background against which inward currents act. Foundational deletion studies implicated TREK-1 in anesthetic sensitivity and TASK channels in volatile-anesthetic immobility, with cholinergic-neuron deletion helping localize the TASK effect to motor circuitry [23,24]. More recent molecular dynamic and functional experiments linked the movement of the TREK cytosolic sensor domain to the stability of the selectivity filter, explaining how phosphorylation and membrane interactions can regulate conductance without changing channel abundance [25]. A membrane-dependent anesthetic mechanism involving phospholipase D2 and phosphatidic acid provides a complementary route to TREK-1 activation [26].

The direction of the pain effect is experimentally testable. A photoswitchable TREK/TRESK inhibitor enabled the reversible control of nociception in freely moving animals; acute inhibition at peripheral endings was sufficient to provoke pain-related responses [27]. For Kv7 channels, pharmacological interference reduced acetaminophen-associated antinociception in an inflammatory model, supporting a peripheral potassium channel contribution [28]. These findings do not mean that every potassium channel opener is analgesic or anesthetic. Subtype distribution, resting voltage, and effects on motor and autonomic neurons determine whether increasing an outward conductance has a useful therapeutic window.

Calcium channels act at both spike generation and synaptic release. Low-threshold CaV3.2 knockdown reduced sensory T-type current and pain behavior in early antisense experiments, establishing a causal preclinical link [29]. In contrast, CaV2.2 is particularly important at presynaptic terminals. Human CaV2.2 structures resolved how ziconotide occupies the extracellular pore and how the auxiliary α2δ-1 subunit moves to accommodate binding [30]. The newer compound C2230 stabilized inactivated CaV2.2; inhibited currents in rodent, marmoset, and human sensory neurons; and reduced spinal transmitter release and pain-like behavior in preclinical models [31]. Clinical efficacy remains a separate requirement.

The α2δ-1 protein cannot be reduced to a passive calcium channel accessory. A pregabalin-binding-site mutation established its requirement for gabapentinoid analgesia [32], while recent experiments linked α2δ-1 to NMDA receptor trafficking and spinal plasticity. The theta-burst stimulation of primary afferents produced long-term potentiation preferentially in excitatory dorsal horn neurons; the genetic deletion of α2δ-1 or disruption of its NMDA receptor interaction prevented this potentiation and associated hypersensitivity [33]. Subsequent work showed that GABAergic and glycinergic inhibition restrains α2δ-1- and mGluR5-dependent NMDA receptor activity at primary-afferent-to-excitatory-neuron synapses [34]. Channel trafficking and inhibitory tone therefore converge on the same synaptic amplification mechanism.

These synaptic experiments also clarify why stimulus pattern matters. Theta-burst input induced potentiation in approximately half of the tested excitatory lamina II neurons, whereas inhibitory neurons showed potentiation less frequently and occasional depression. Nerve injury increased the prevalence of potentiation selectively in the excitatory population [33]. The aggregate dorsal horn response would conceal that asymmetry. In a later disinhibition study, sensory-neuron *Grin1* deletion separated presynaptic from postsynaptic NMDA effects, while α2δ-1 deletion removed a broader component [34]. Such intersectional experiments are more informative than measuring total receptor proteins because they locate the relevant molecular interaction within a specific synaptic connection.

### 2.3. Transduction Channels Connect Membrane State to Stimulus Selectivity

Recent PIEZO2 studies revise the simple division between innocuous touch and mechanical pain. Nociceptor-specific deletion protected against mechanical sensitization in experimental osteoarthritis and inflammatory joint injury [35]. Conversely, mutations relieving voltage-dependent PIEZO2 block strongly sensitized nociceptors and could drive ongoing activity [36]. Membrane depolarization can therefore alter not only the spike threshold but the force sensitivity of the transduction process itself. Human microneurography also identifies thickly myelinated high-threshold mechanoreceptors that encode painful indentation, cautioning against identifying nociception exclusively with slowly conducting fibers [37].

Lipid and accessory-protein regulation creates additional intervention points. Phosphatidic acid inhibited PIEZO2 more selectively than PIEZO1 in the tested systems, linking phospholipase D activity to mechanical sensitivity [38]. MDFIC2 shifted PIEZO gating toward higher required forces; its expression decreased after nerve injury, and AAV-mediated restoration attenuated mechanical allodynia in mice [39]. TRPV1 structures likewise showed that endogenous lipids occupy a regulatory pocket whose occupancy changes channel activation [40]. The same lipid mediator need not have the same effect on every channel, so membrane remodeling should be understood as target-specific regulation rather than generalized membrane fluidization.

**Figure 1:**
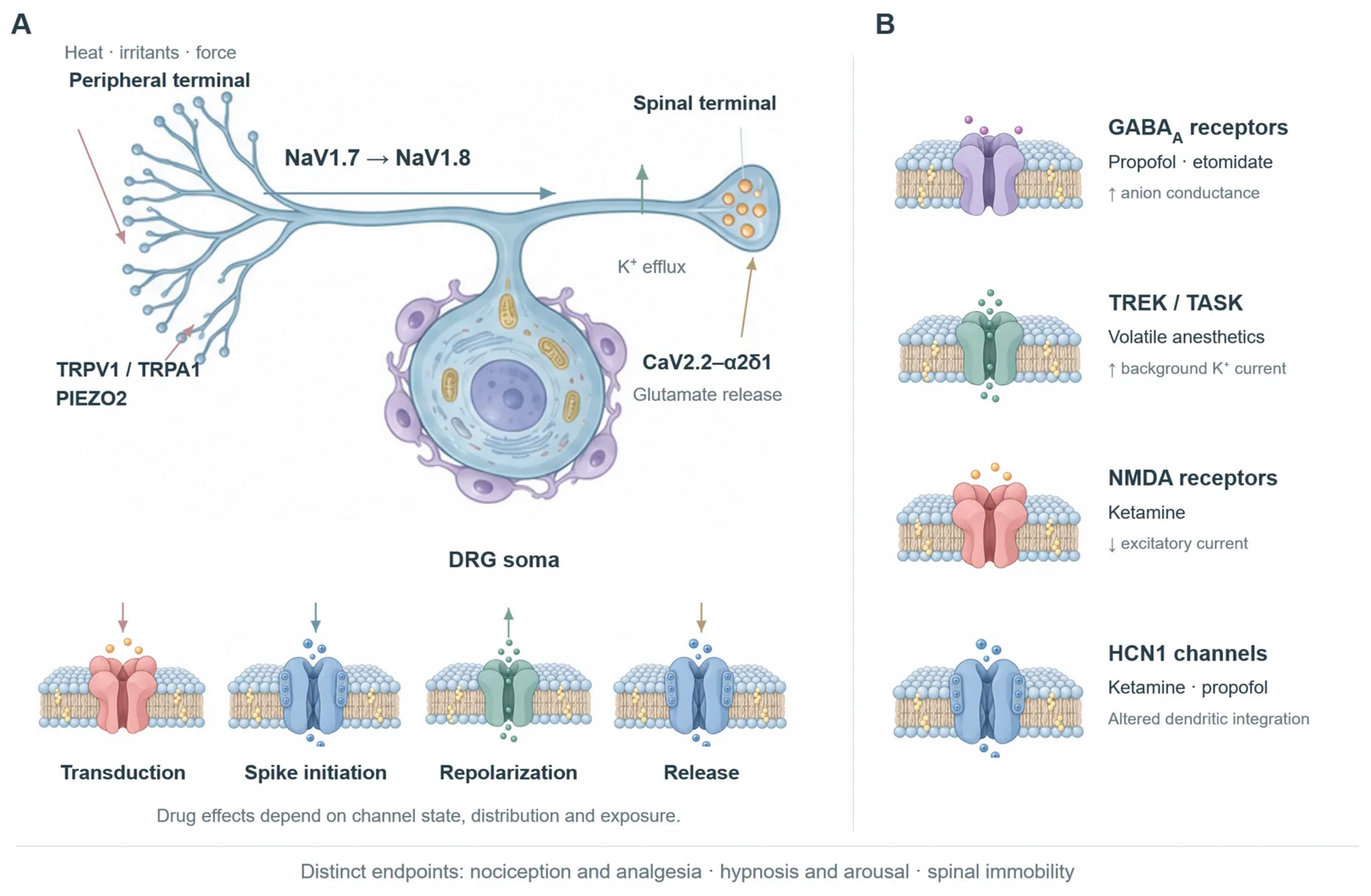
Molecular operations linking sensory input to anesthetic responses. (**A**) A pseudounipolar sensory neuron illustrates the distinction between peripheral transduction, sodium-channel-dependent firing, potassium-mediated repolarization, and calcium-dependent spinal transmitter release. The membrane strip groups functions and does not imply identical subcellular localization. Recent human recordings show that NaV1.8 inhibition can narrow spikes without abolishing repetitive firing, while PIEZO2 studies connect membrane voltage to mechanical transduction. (**B**) Selected anesthetic targets. GABAA potentiation, TREK/TASK activation, NMDA inhibition, and HCN1 modulation have different cellular consequences and drug dependencies; they are not shown as equal contributors to all anesthetic endpoints. DRG, dorsal root ganglion. In (**A**), arrows indicate signal propagation, ion movement, or the labeled structures. In (**B**), ↑ and ↓ indicate increases and decreases, respectively. Colors distinguish schematic components and have no quantitative meaning.

TRP channel interventions also require modality and safety distinctions. Sigma-1 receptor antagonism reduced TRPA1 surface expression and function and prevented oxaliplatin-associated hypersensitivity in a mouse model [41]. GLP-1-derived peptides inhibited selected modes of TRPV1 activation and reduced pain behavior without the hyperthermia associated with some other TRPV1 inhibitors in the tested mice [42]. These are promising mechanistic separations, not established clinical alternatives. Figure 1 summarizes the different operations performed by transducer channels, spike-generating conductances, presynaptic machinery, and anesthetic receptor targets.

PIEZO2 additionally illustrates how a channel can participate in both protective sensation and pathological amplification. Joint-injury experiments implicated a subset of PIEZO2/NTRK1-coexpressing nociceptors, distinct from treating all mechanically sensitive neurons as one therapeutic population [35]. A treatment aimed at mechanical allodynia would need to distinguish these functions from ordinary touch and proprioception. Accessory-protein modulation is attractive because it may change sensitivity rather than remove mechanotransduction altogether, but MDFIC2 restoration also reduced mechanical sensitivity in the tested mice [39]. Preserving everyday tactile and motor coordination functions therefore remains a necessary experimental endpoint, not an assumed consequence of peripheral restriction.

## 3. Inhibitory Receptors and Circuits That Determine Anesthetic Responses

### 3.1. The Receptor Structure Explains Drug Sensitivity but Not the Entire Anesthetic State

Evaluating the β3 N265M GABAA receptor mutation remains a decisive experiment because it markedly reduced selected propofol and etomidate responses in mice, connecting receptor pharmacology to behavior [43]. Structural work subsequently identified distinct and overlapping transmembrane sites for propofol, etomidate, and phenobarbital [44]. Recent studies add a further complication: the same set of GABAA subunits can assemble in different stoichiometries and arrangements, creating or removing intersubunit ligand-binding sites. Receptor composition is therefore more than the presence or absence of an individual transcript [45].

Structural and functional experiments now separate binding from gating more directly. Noncanonical amino-acid substitutions identified a main-chain hydrogen bond in the β2 M2–M3 linker that helps keep the unliganded receptor closed; disrupting the analogous bond in α1 did not have the same effect [46]. Quinazolinone-bound structures showed a deeper occupation of sites also targeted by propofol and etomidate and widening of the extracellular pore [47]. In 2026, the structures of human α5β3 receptors revealed predominantly 2α:3β assembly and results consistent with conformational selection during etomidate potentiation [48]. These observations refine molecular models but do not establish which receptor assemblies dominate a particular anesthetic endpoint in vivo.

The assembly problem has direct consequences for interpreting sequencing data. The detection of α4, β3, and δ transcripts does not establish how many copies of each subunit occupy an individual receptor or whether γ2-containing receptors coexist in the same cell. Recombinant structural studies resolved alternative arrangements from these subunit combinations and showed changes in responsiveness to physiological and synthetic modulators [45]. A transcript-based anesthetic sensitivity score would therefore omit receptor assembly, membrane trafficking, and basal gating. Linking an identified neuronal class to a drug response requires functional recording or protein-level evidence in addition to an expression signature, particularly when several receptor populations contribute to the recorded current.

HCN1 provides a parallel example beyond GABAA receptors. Earlier knockout studies connected HCN1 to ketamine- and propofol-induced loss of righting, and forebrain-restricted deletion localized part of ketamine sensitivity to excitatory neurons [49,50]. A 2024 structural study identified propofol binding between HCN1 S5 and S6 helices and demonstrated the restoration of voltage-dependent closing in two epilepsy-associated variants [51]. Because HCN current contributes to both resting voltage and dendritic filtering, its inhibition can hyperpolarize cells while increasing synaptic integration. The mean firing rate alone cannot capture this change in input processing.

Ketamine also illustrates concentration-dependent molecular action. The cryo-EM structures of human NMDA receptors placed S-ketamine between the channel gate and selectivity filter, with mutagenesis supporting functionally important contacts [52]. A later study reported a distinct, low-concentration allosteric interaction at membrane-accessible sites that preferentially reduced tonic activation while sparing brief synaptic-like responses [53]. These findings should not be collapsed into one mechanism covering analgesia, hypnosis, and antidepressant effects. Drug concentration, receptor activation history, and the behavioral endpoint determine which molecular observation is relevant.

### 3.2. Chloride Homeostasis Connects Receptor Activation to Neuronal Outcome

GABAA and glycine receptor activation depends on ionic driving force as well as channel opening. Original KCC2 experiments connected nerve injury to a depolarized anion reversal potential in spinal neurons and showed that reducing chloride extrusion can produce hypersensitivity [54]. GlyR α3 phosphorylation by prostaglandin E2 provided a distinct receptor-level mechanism of inflammatory disinhibition [55]. These mechanisms must remain separate: restoring receptor number or potentiating channel opening may not restore the original inhibitory response when the chloride gradient has changed. Depolarizing responses can also remain shunting, so depolarization alone is insufficient evidence of net excitation.

Recent anesthesia experiments extend KCC2 regulation beyond spinal pain. In mice, the phosphorylation-dependent recruitment of the ubiquitin ligase Fbxl4 promoted KCC2 degradation in ventral posteromedial thalamic neurons, facilitating emergence from anesthesia [56]. Follow-up experiments implicated VCP and FAF1 in delivering ubiquitinated KCC2 to the proteasome; interfering with this process increased anesthetic effects [57]. The same reduction in chloride extrusion can therefore facilitate arousal in one circuit and pathological sensory amplification in another. These studies identify a regional mechanism in experimental animals, not a recommendation for systemic KCC2 inhibition during clinical recovery.

Inhibitory synapse structure is another independent variable. Time-resolved studies found the microglial removal of inhibitory dorsal horn synapses before excitatory synapse removal after nerve injury [58]. More selective experiments linked the ApoE/C1q-associated pruning of glycinergic terminals to the disinhibition of PKCγ interneurons and increased access of low-threshold Aβ input to pain pathways [59]. An observed reduction in inhibitory current could thus arise from altered chloride transport, receptor phosphorylation, fewer synapses, or changes in release. Identifying the responsible process is essential before choosing a restorative treatment.

### 3.3. Arousal and Antinociception Recruit Partially Separable Populations

Cell-specific manipulations have replaced the idea of uniform brain suppression with experimentally defined transitions. Population activity of paraventricular thalamic glutamatergic neurons decreased before propofol-induced loss of consciousness; optogenetic stimulation of calretinin-expressing neurons in this region during continuous infusion restored cortical activation and behavioral arousal within seconds [2]. Orexinergic terminals in the basal forebrain and locus coeruleus promoted emergence from isoflurane in rats, while locus-coeruleus-to-paraventricular-thalamus stimulation accelerated recovery in mice [60,61]. These manipulations establish causal influence on recovery, but a circuit that overcomes anesthesia is not necessarily the initial molecular site of anesthetic action.

The thalamic study is informative because it tested transitions in both directions under controlled propofol administration. Chemogenetic inhibition shortened the mean time to loss of righting from 176 to 127 s and prolonged return from 1568 to 3126 s; activation produced the opposite pattern [2]. Brief stimulation during maintained infusion further reduced the possibility that the effect simply reflected faster drug elimination. Nevertheless, righting is a behavioral correlate of arousal that depends on motor output. Combining it with cortical recordings strengthens the interpretation without establishing the restoration of every component of consciousness, memory formation, or nociceptive awareness.

The neurotransmitter label alone does not predict circuit direction. Ventrolateral periaqueductal gray GABAergic neurons promoted arousal through a cortico-midbrain circuit, whereas supramammillary glutamatergic neurons and their medial septal projection also promoted recovery under sevoflurane [62,63]. Orexinergic projections to substantia innominata combined arousal-promoting and antinociceptive effects, predominantly through orexin receptor 2 in the reported experiments [64]. Projection identity and postsynaptic partners explain these effects more usefully than a global statement that inhibition produces anesthesia and excitation reverses it.

Anesthetic-recruited central amygdala neurons provide a direct bridge to pain. The initial study identified a GABAergic ensemble whose activation suppressed nocifensive behavior [65]. A 2025 investigation using a different activity-tagging strategy confirmed antinociception but found weaker effects at eight weeks than at two weeks after nerve injury, alongside altered excitability and KCC2 expression in other amygdala neurons [3]. Propofol also reduced pain-associated anxiety-like behavior through the GABAA β3-dependent inhibition of hypothalamic CRH neurons in mice, an endpoint distinct from the nociceptive threshold [66]. These observations argue for measuring sensory, affective, and arousal outcomes separately.

Descending control can even reverse the apparent meaning of a molecular mediator. Morphine-responsive BDNF-expressing rostral ventromedial medullary neurons recruited inhibitory spinal galanin neurons and mediated mechanical antinociception [67]. BDNF is therefore not intrinsically pronociceptive; its source and recipient determine the effect. Longitudinal imaging likewise showed different spinal output reorganization after acute capsaicin sensitization and chronic nerve injury [68]. Finally, fly studies demonstrated fragmented neural ensembles and the synapse-selective disruption of presynaptic release under isoflurane, rather than complete neuronal silence [69,70]. Their transferable principle is altered coordination, not an assumption that insect synaptic classes map directly onto mammalian circuits. Within the broader pain framework, supraspinal circuits should be considered together with peripheral sensitization, spinal disinhibition, and neuroimmune signaling. Peripheral and spinal mechanisms shape the input reaching higher centers, while descending pathways modify its transmission. The reduced antinociceptive effect of activating anesthetic-responsive amygdala neurons at a later stage of nerve injury illustrates that central pain control is itself state-dependent [3]. Changes in the recruitment or effectiveness of endogenous inhibitory circuits may therefore contribute to persistent pain alongside peripheral and spinal abnormalities. Nevertheless, the thalamic and orexinergic studies discussed above establish distinct effects on arousal or antinociception and should not all be interpreted as evidence of chronic pain maintenance. Their integration requires circuit-specific tests across injury stages, with sensory, affective, and arousal outcomes measured separately.

## 4. Neuroimmune Signaling and Persistent Reprogramming After Injury

### 4.1. Immune Cells Can Sustain Pain or Enable Recovery

The microglial P2X4–BDNF pathway established that an immune-cell response can change neuronal chloride regulation and sensory behavior [71,72]. Recent profiling shows why activated microglia should not be treated as a single state. After nerve injury, spinal microglial transcription differed by sex and time, with male-enriched inflammatory populations and prominent chronic *Apoe* expression [73]. These findings complement, rather than replace, measurements of neuronal inhibition: a transcriptional state identifies candidate effectors but does not establish their electrical consequence.

Sex-dependent mechanisms are more specific than a binary claim that microglia matter only in males. Pannexin-1 supported microglial VEGF-A release in male rodents, whereas female-derived CD8+ T cells released leptin through a pannexin-dependent pathway that sustained allodynia [74]. In contrast, manipulating microglial 4E-BP1-dependent translation altered mechanical hypersensitivity in both sexes, although spontaneous pain effects differed [75]. These results extend the earlier demonstration of alternative immune routes to comparable behavioral hypersensitivity and show that sex dependence must be assigned to a defined pathway and endpoint [76].

Some glial responses actively limit pain. CD11c-expressing spinal microglia appeared after hypersensitivity developed, and their IGF1 signaling was required for spontaneous recovery; depletion after remission caused relapse [77]. Astrocytic GPR37L1 provided another protective mechanism: its activation enhanced GLT-1 activity, reduced excitatory synaptic currents, and alleviated neuropathic pain, whereas deficiency impaired recovery [78]. Broadly suppressing glial activity could therefore inhibit both pathogenic and restorative processes. The timing of intervention may determine whether an immune target offers benefit.

BDNF illustrates the contextual framework of this review. Microglial BDNF can weaken chloride-dependent inhibition in spinal lamina I neurons in neuropathic pain models [67,72]. In contrast, morphine-responsive BDNF-expressing neurons in the rostral ventromedial medulla recruit inhibitory spinal galanin-positive neurons through a BDNF/TrkB-dependent mechanism and mediate mechanical antinociception in mice [67,72]. These findings do not assign a uniformly harmful or beneficial role to BDNF. Rather, they show why molecular identity must be interpreted together with the source and recipient cells, circuit organization, and experimental or pathological context. Consequently, a bulk increase in BDNF cannot by itself identify the physiological abnormality or justify a direction of therapeutic intervention.

### 4.2. Peripheral Signaling Is Bidirectional and Injury-Specific

Peripheral cytokines act on defined sensory populations. The IL-23–macrophage IL-17A pathway produced mechanical hypersensitivity in female mice through TRPV1-expressing neurons and neuronal estrogen receptor α [79]. Human, macaque, and mouse recordings subsequently showed sex-selective nociceptor sensitization by prolactin in females and orexin B in males [80]. This cross-species functional evidence is stronger than receptor expression alone, although donor numbers, hormonal context, and disease state still constrain generalization to patients.

Different injuries recruit different signals. A transcriptomic comparison of skin incision, zymosan inflammation, and ultraviolet injury identified distinct immune programs and found that thrombospondin-1 could inhibit nociceptor sensitization [81]. In experimental gout, CXCL5 activated neuronal CXCR2 and TRPA1, linking immune signaling to electrical hyperexcitability and neuropeptide-dependent inflammation [82]. In contrast, STING–type I interferon signaling rapidly suppressed excitability in mouse, monkey, and human nociceptors, and STING agonists reduced bone cancer pain through combined neuronal, immune, and skeletal effects in mice [83,84]. The term inflammation therefore does not specify the direction of neuronal modulation.

Nociceptors also regulate the tissue being injured. The ablation of NaV1.8-lineage neurons impaired wound repair and muscle regeneration, with CGRP–RAMP1 signaling coordinating neutrophil and macrophage responses [85]. In endometriosis models, however, CGRP–macrophage communication supported lesion growth and pain, and blocking this axis reduced both [86]. These findings have a practical perioperative implication: the consequences of eliminating a sensory population cannot be assumed to match transient channel inhibition. They raise questions about tissue repair that should be tested directly, without implying that clinical NaV1.8 inhibitors necessarily reproduce neuronal-ablation phenotypes.

The contrasting CGRP studies identify different macrophage outcomes rather than an unexplained contradiction. During acute repair, neuronal signaling promoted the clearance of dying cells and a pro-repair phenotype. In endometriosis lesions, CGRP-exposed macrophages showed impaired efferocytosis and supported endometrial-cell growth [85,86]. The tissue environment and the duration of signaling are therefore part of the mechanism. For a postoperative analgesic, wound closure, infection, and recovery should be evaluated alongside pain scores if the intervention substantially changes neuroimmune communication. An improvement in mechanical withdrawal behavior alone would not detect an unfavorable effect on these other sensory-neuron functions. These findings suggest that the transition to persistent pain depends partly on whether injury responses successfully engage recovery mechanisms. Acute neuroimmune signaling can support defense and tissue repair, whereas persistent tissue pathology, impaired clearance, or insufficient recruitment of protective glial programs may favor continued sensitization [77,78,85,86]. A useful organizing model is therefore acute injury followed by inadequate resolution, sustained neuronal–immune amplification, and the stabilization of altered excitability through transcriptional or epigenetic regulation. However, the studies reviewed here establish individual components of this model rather than a single obligatory sequence. Recovery and pathogenic signaling may coexist, and their relative contributions depend on tissue, sex, and injury stage. This distinction connects the immune mechanisms discussed above with the persistent regulatory changes considered below.

### 4.3. Chromatin and RNA Regulation Stabilize Altered Excitability

Persistent pain can reflect changes in the conductances available to a neuron rather than continuing exposure to the initiating mediator. G9a-dependent H3K9 dimethylation suppressed multiple potassium channel genes after nerve injury, and sensory-neuron deletion prevented this repression in foundational experiments [87]. More recent work identified the loss of the ZNF382-mediated silencing of *Cxcl13*, including the disruption of a repressive chromatin complex at a silencer–promoter loop [88]. These mechanisms operate at different genomic sites and should not be described simply as globally increased or decreased epigenetic activity.

RNA modification and metabolic signaling add further layers. METTL14-dependent m6A modification stabilized GluN2A-related signaling through IGF2BP2 in chemotherapy-associated neuropathic pain experiments [89]. In 2025, amphiregulin–EGFR signaling was linked to PKM2-dependent glycolysis, increased histone lactylation, and pronociceptive gene expression; intervention at several points reduced neuronal hyperexcitability and pain behavior in mice [90]. Such multi-step perturbation strengthens a causal model, but the breadth of the affected pathways also creates selectivity concerns. A systemic inhibitor of a chromatin or metabolic regulator may influence repair, immunity, and other organs independently of nociception.

Cell identity itself changes after injury. ATF3-dependent transcriptional reprogramming suppressed mature sensory-neuron features while supporting regeneration, and later single-cell analysis resolved temporally changing injury-associated populations [91,92]. The apparent disappearance of a marker-defined class can therefore reflect state transition rather than cell death. Senescence provides another candidate persistent state: the iPain atlas and subsequent electrophysiological work identified senescence-associated sensory neurons, including high-firing phenotypes, and senolytic interventions improved nociceptive behavior in mice [93,94]. These results do not yet establish the safety or efficacy of senolysis for human chronic pain. Together, these mechanisms support a distinction between the persistence of an initiating stimulus and consolidation of an altered cellular state. Chromatin regulation, RNA modification, metabolic reprogramming, and senescence-associated changes can alter the machinery controlling excitability, potentially sustaining hypersensitivity beyond the initiating stimulus [87,88,89,90,91,92,93,94]. Here, pathological consolidation refers to the stabilization of an abnormal state, not necessarily irreversibility or complete independence from ongoing peripheral input. Its contribution must be established separately in each model. Combined with evidence for active glial recovery programs [77,78], this perspective suggests that chronic pain may involve both persistent excitation and failure to restore normal regulation. A corresponding therapeutic question is whether an intervention transiently suppresses signaling or durably restores the processes required for resolution.

**Figure 2:**
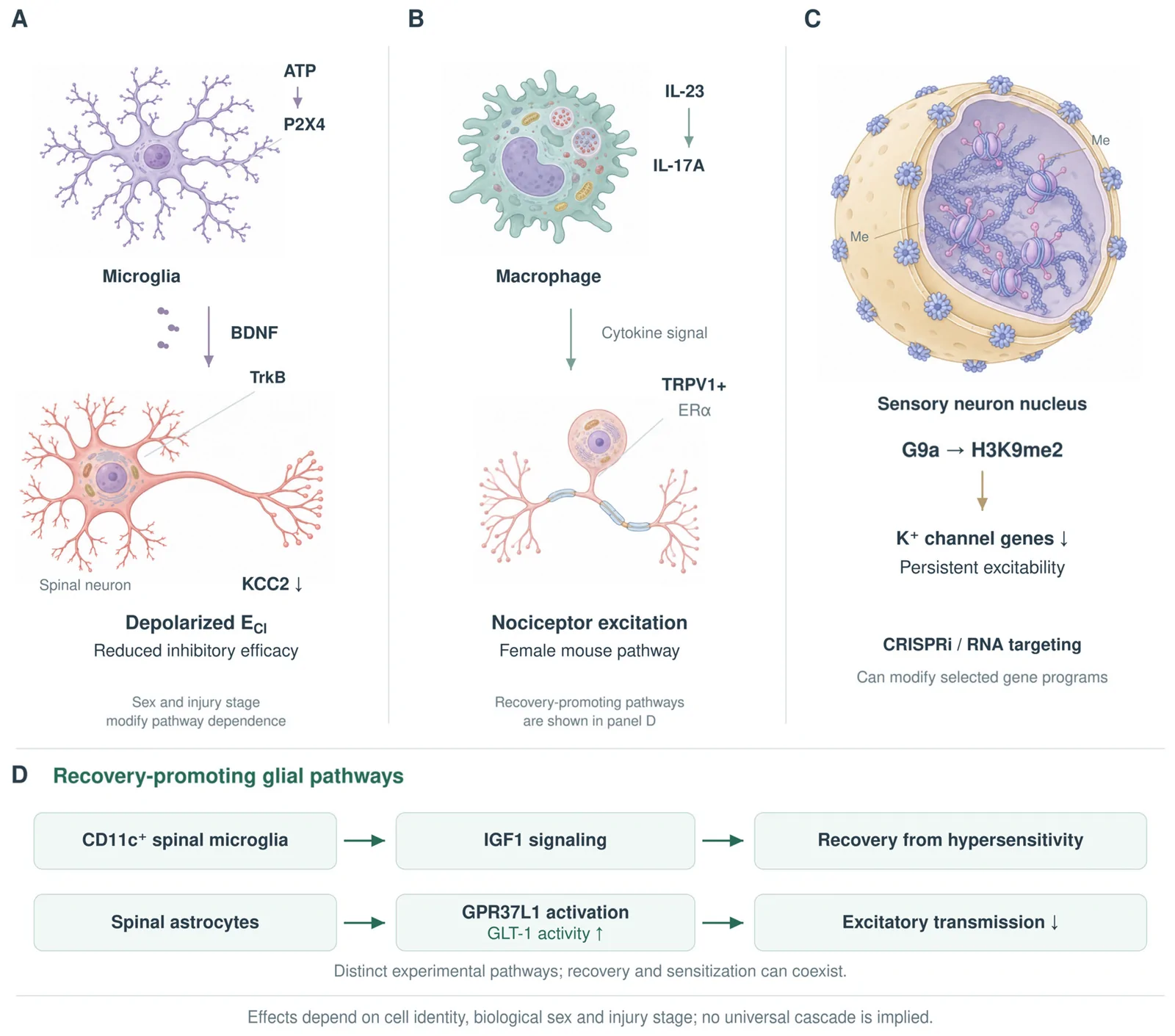
Neuroimmune and transcriptional mechanisms promoting persistent pain or recovery. (**A**) Microglial P2X4–BDNF signaling can reduce neuronal KCC2-dependent chloride extrusion, depolarize the chloride equilibrium potential, and weaken spinal inhibition. Pathway dependence varies with sex and injury stage. (**B**) The IL-23–macrophage IL-17A pathway acts through TRPV1-expressing sensory neurons and requires neuronal ERα in the tested female mice. (**C**) G9a-dependent H3K9 dimethylation illustrates the persistent repression of potassium channel genes. Targeted transcriptional and RNA interventions can modify selected gene programs, as discussed in the text. (**D**) Recovery-promoting pathways include CD11c-positive spinal microglial IGF1 signaling and astrocytic GPR37L1-dependent enhancement in GLT-1 activity, supporting recovery from hypersensitivity and reduced excitatory synaptic transmission, respectively. These pathways represent parallel examples from distinct experimental models, not a single universally established cascade. BDNF, brain-derived neurotrophic factor; TrkB, tropomyosin receptor kinase B; KCC2, potassium–chloride cotransporter 2; ECl, chloride equilibrium potential; ERα, estrogen receptor α; H3K9me2, histone H3 lysine 9 dimethylation; Me, methylation; IGF1, insulin-like growth factor 1; GLT-1, glutamate transporter 1; CRISPRi, CRISPR interference. Connecting arrows indicate the interactions or downstream effects depicted; ↑ and ↓ beside labels indicate increases and decreases, respectively. Colors distinguish schematic components and do not represent quantitative values.

Targeted repression may be more selective than global chromatin manipulation. The intrathecal AAV delivery of CRISPR-dCas9 or zinc-finger repressors reduced NaV1.7 expression and hypersensitivity in mouse models [95]. Trafficking-directed NaV1.7 constructs and MDFIC2 restoration offer mechanistically different gene-delivery strategies [17,39]. Protective sensation, durability, vector distribution, and the ability to stop treatment become central safety questions when modulation persists for months. Figure 2 places pain-promoting immune signaling and persistent gene regulation alongside recovery-promoting glial pathways while preserving their distinct sites, experimental contexts, and timescales.

## 5. Human Cellular Models and Clinical Translation

### 5.1. Atlases Become More Useful When Linked to Function

Human sensory atlases have changed target prioritization by revealing both conserved classes and species-specific expression. The spatial profiling of the DRG from eight donors identified twelve sensory-neuron clusters, including five C-nociceptor groups [96]. Human nuclear profiling independently identified classes with imperfect correspondence to mouse populations [97]. Harmonization across 31 datasets and six species subsequently organized eighteen neuronal and eleven nonneuronal types while preserving important differences in channel and neuropeptide expression [98]. A familiar cluster name is consequently not sufficient evidence that a drug target is expressed at a comparable level or location across species.

The deep sequencing of individually isolated human neuronal somata provides an important complement to nuclear surveys. One study detected more than 9000 genes per neuron on average, resolved sixteen neuronal types, and combined spatial validation with molecularly informed microneurography [99]. Somatic and nuclear measurements sample different RNA compartments and differ in sensitivity, so disagreement about low-abundance channels can be technical as well as biological. Conversely, better transcript detection does not establish a functional protein at the peripheral ending or spinal terminal. Target selection benefits the most when the same proposed subtype is supported by independent anatomical, molecular, and physiological evidence.

The spinal cord now has complementary human resources. An adult cellular taxonomy identified 35 neuronal and 29 glial clusters, while a separate spatially resolved study of nine donors classified 21 neuronal subclusters [100,101]. The different counts reflect sampling and classification choices and should not be presented as incompatible anatomical inventories. Human glial atlases further document species and sex differences [102]. Cross-species dorsal horn transcriptomic and chromatin integration linked chronic-pain-associated regulatory variation to particular neuronal populations, offering a way to move from noncoding association to a testable cellular hypothesis [103]. Trigeminal ganglion atlases provide a comparable framework for headache mechanisms [104].

Regulatory maps are particularly useful because many associated variants lie outside the coding sequence. An enrichment in pain-associated variation in subtype-specific accessible chromatin can nominate the cells in which a locus operates, but it does not identify the causal nucleotide or prove which nearby gene is regulated. The dorsal horn study combined a rhesus neuronal atlas, human and mouse integration, spatial localization, and mouse chromatin accessibility to constrain that interpretation [103]. Subsequent work still needs allele-specific regulatory assays and perturbation in a relevant cell background. Assigning a noncoding variant to the nearest ion channel gene without these intermediate tests can produce a plausible but incorrect mechanism.

Functional anchoring is particularly important for sensory subtypes that lack a clear molecular label. A 2026 study combined pig Patch-seq, cross-species integration, and human microneurography to identify OSMR and SST as markers associated with mechanically insensitive C fibers. Oncostatin M selectively modulated these fibers in healthy volunteers [105]. This sequence is more informative than annotating a cluster from one marker: electrophysiology established a functional class, transcriptomics nominated receptors, and human testing assessed a predicted response. It also shows why SST expression cannot automatically be interpreted as identifying an inhibitory neuron in every anatomical region.

The distinction between human recording approaches is equally important. Dissociated DRG patch clamp measures somatic membrane properties under experimentally controlled conditions, whereas microneurography samples action potentials in intact peripheral afferents responding through their native endings. In a 2026 mechanical pain study, robotic indentation and single-unit recording distinguished high-threshold myelinated and unmyelinated afferents; the selective microstimulation of one myelinated high-threshold unit evoked localized sharp-stinging pain [37]. This directly relates identified afferent activity to a perceptual report. It does not establish which molecular inhibitor would suppress that percept safely, but it provides a human functional endpoint against which a proposed target could be tested.

Stem-cell models offer experimental access unavailable in adult donors. A developing human DRG atlas guided organoid differentiation and identified a human-enriched NTRK3+/DCC+ nociceptor population [106]. Four-part human ascending sensory assembloids connected somatosensory, spinal, thalamic, and cortical components; *SCN9A* loss disrupted synchrony, while a gain-of-function variant produced hypersynchrony [107]. These systems enable controlled genetic perturbation and interregional recording, but their maturity, connectivity, and exposure conditions differ from those of an adult patient. Synchronized activity in an assembloid is not evidence of subjective pain or a validated substitute for clinical analgesia.

### 5.2. Genetic Association Requires a Mechanistic Intermediate

The SLC45A4 study exemplifies a productive follow-up of a human association. A pain intensity locus was connected to a selective polyamine transporter, cryo-EM structure, and reduced C-polymodal nociceptor excitability in knockout mice. Heat and algogen responses changed, while mechanical sensitivity was preserved, narrowing the functional phenotype [7]. By comparison, a genome-wide association with chronic postsurgical pain at *GLRA3* is biologically plausible but does not yet establish the relevant variant effect or a genotype-guided treatment [108]. Gene nomination and therapeutic validation remain separate achievements.

Large-scale studies also reveal shared liability rather than a single peripheral defect. Genomic modeling across 24 chronic pain conditions identified broad shared genetic risk and a more specific musculoskeletal component [109]. Plasma proteomics associated numerous immune and metabolic proteins with pain and future spread, but circulating abundance does not establish action at a particular synapse [110]. These resources can prioritize hypotheses and patient strata. They cannot replace perturbation, external predictive validation, or a demonstration that changing the proposed mediator improves an outcome.

Pharmacokinetic genetics answer a different question. *CYP2D6* variation can alter the conversion of codeine or tramadol to active metabolites without changing a neuron’s response at a given drug concentration. CPIC recommendations distinguish these actionable associations from the more limited evidence for *OPRM1*- or *COMT*-guided prescribing [111]. Similarly, an association with anesthetic dose may reflect clearance, co-medication, age, or administration practice rather than receptor sensitivity. Concentration–response phenotyping and the explicit separation of arousal, movement, memory, and analgesia are needed before assigning an excitability mechanism to a clinical genetic association.

### 5.3. NaV1.8 Trials Define Both Progress and Remaining Limitations

In phase 2 postoperative studies, the highest VX-548 regimen improved the 48 h time-weighted sum of pain intensity differences compared with placebo after abdominoplasty and bunionectomy. Between-group SPID48 differences were 37.8 and 36.8 units, respectively [112]. These are integrated pain-over-time outcomes, not differences of nearly forty points on a ten-point scale. Subsequent phase 3 trials included 1118 and 1073 treated participants and found placebo-adjusted SPID48 differences of 48.4 and 29.3. Neither demonstrated the prespecified superiority over hydrocodone–acetaminophen [1]. Failure to establish superiority does not establish equivalence.

A separate single-arm study treated 256 participants with surgical or nonsurgical acute pain for up to fourteen days; 83.2% were classified as rating effectiveness as good, very good, or excellent in the end-of-treatment analysis [113]. Without a concurrent comparator, these ratings cannot estimate comparative efficacy or disentangle treatment effects from natural recovery. Together, the trials establish a clinically effective nonopioid mechanism in studied acute pain settings while leaving persistent pain prevention, optimal combinations, and comparative effectiveness in other populations unresolved. The residual firing observed in human neurons provides a plausible mechanistic explanation for incomplete analgesia, but linking an individual cellular phenotype to a patient’s response remains an untested inference [4].

**Figure 3:**
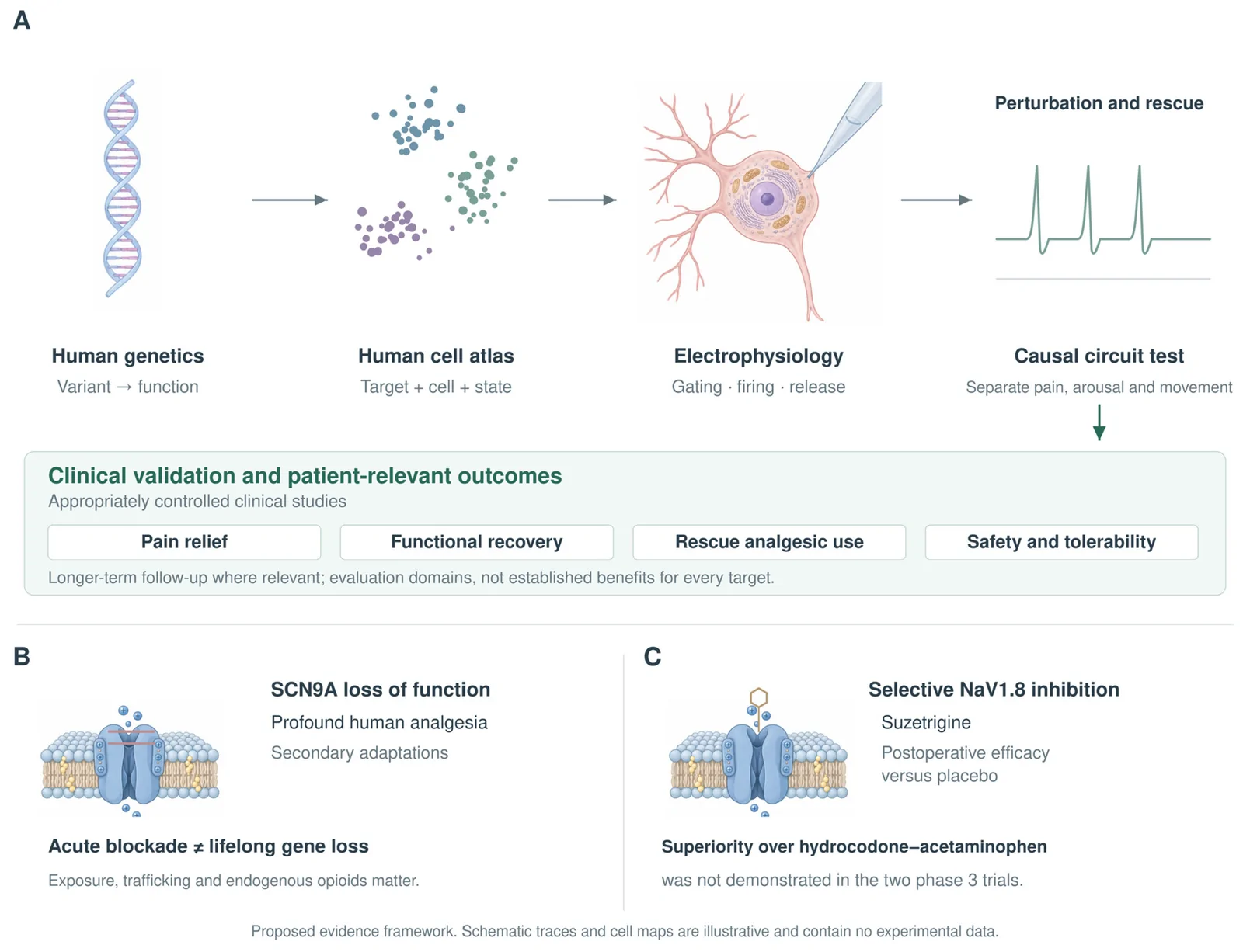
The evidence required to translate a molecular target into analgesia. (**A**) Human genetics and cellular atlases nominate targets, while electrophysiology and causal perturbation establish function. Clinical validation is shown as the final stage, with patient-relevant evaluation domains including pain relief, functional recovery, rescue analgesic use, and safety and tolerability. Longer-term follow-up is needed where relevant. These domains are evaluation priorities, not benefits already demonstrated for every target. (**B**) NaV1.7 illustrates why lifelong gene loss and acute pharmacological inhibition need not produce identical outcomes. Drug exposure, channel trafficking, and secondary adaptations, including endogenous opioid mechanisms, require consideration. (**C**) Selective NaV1.8 inhibition with suzetrigine demonstrated postoperative efficacy versus placebo, but superiority over hydrocodone–acetaminophen was not demonstrated in the two pivotal phase 3 trials. Cell maps and voltage traces are schematic illustrations, not experimental data.

The trial sequence should also be distinguished from a claim of demonstrated opioid sparing under routine multimodal care. The pivotal studies compared assigned treatments in defined surgical models; the magnitude of benefit when a NaV1.8 inhibitor is added to regional anesthesia, anti-inflammatory drugs, and other perioperative measures requires its own comparison [1]. Likewise, selective peripheral pharmacology supports a rationale for avoiding direct central opioid-receptor effects, but it cannot exclude all adverse events, drug interactions, or rare harms. Follow-up beyond the acute treatment period is necessary to determine whether lower early pain changes persistent pain risk rather than merely improving symptoms while the drug is present. Figure 3 summarizes the evidence linking molecular target discovery to clinical analgesic validation.

## 6. Conclusions and Priorities for Perioperative Translation

The next translational step is to define which component of excitability needs correction in a particular condition. A channel mutation may require a gating-specific intervention; inflammatory sensitization may be better addressed at a receptor or accessory protein; persistent injury states may require transcriptional modulation. Experiments should measure the property implied by the proposed mechanism. The threshold alone is inadequate for a drug acting on spike width or transmitter release. RNA abundance is inadequate for altered surface trafficking. Conventional whole-cell recordings that impose intracellular chloride are inadequate for establishing a native chloride-gradient defect. Appropriate assays reduce the risk of selecting a treatment on the basis of an indirect surrogate.

Human validation should occur before a mechanism becomes entrenched in a therapeutic program. Adult donor neurons test pharmacology in a relevant conductance background, isogenic induced neurons isolate variant effects, and assembloids test propagation across connected populations. Each has limitations: donor-level biological replication remains essential, cultured cells may change state, and developmental models may not express mature channels or injury responses. Convergence across models is more persuasive than adding many assays to the same preparation. Structural selectivity should be accompanied by clinically meaningful free-drug exposure and physiological stimulus protocols, as the changing interpretation of NaV1.8 state dependence demonstrates. A practical next step is to study molecular target engagement, cellular physiology, circuit activity, and clinically relevant outcomes within a shared experimental or clinical context. For precision anesthesia, this requires distinguishing pharmacokinetic variability from target sensitivity and specifying whether the intended benefit concerns hypnosis, amnesia, immobility, analgesia, or recovery. Such integrated studies would help determine which mechanistic differences are sufficiently reproducible and clinically informative to guide individualized treatment.

For anesthesia, molecular target validation should be linked to a defined behavioral component rather than a generic measure of anesthetic depth. Arousal-promoting stimulation can overcome one component of drug action without reversing amnesia, nociceptive processing, or autonomic effects. For analgesia, withdrawal thresholds, spontaneous pain, affective behavior, functional recovery, and tissue healing should not be treated as interchangeable. Sex and injury stage require prospective consideration because they can change both the responsible cell population and the direction of its contribution. Studies of persistent gene modulation should additionally test protective sensation and the consequences of treatment withdrawal or reversal.

The central message of this review is that a therapeutic target should be defined by its molecular identity, the cells in which it acts, the circuit it influences, the injury or disease stage, and the physiological disturbance to be corrected. The same mediator can support sensitization or recovery in different settings; its abundance alone cannot establish the direction of its functional effect. For chronic pain, persistent symptoms may reflect both continuing pathological input and the stabilization of altered neuronal and neuroimmune states. Treatment may therefore require the restoration of inhibitory control and recovery processes, as well as the suppression of excessive excitation. This framework does not imply a universal mechanism across pain conditions or anesthetic endpoints. Rather, it requires a context-specific chain of evidence linking molecular intervention to a defined physiological correction and, ultimately, a clinically meaningful benefit.

## Data Availability

No new datasets were generated or analyzed for this review. The published sources supporting the discussion are cited in the manuscript and listed in the References.
